# Effect of a restrictive transfusion strategy on transfusion-attributable severe acute complications and costs in the US ICUs: a model simulation

**DOI:** 10.1186/1472-6963-7-138

**Published:** 2007-08-31

**Authors:** Marya D Zilberberg, Andrew F Shorr

**Affiliations:** 1 University of Massachusetts, 715 North Pleasant Street, Amherst, MA, 01003, USA; 2 Washington Hospital Center, 110 Irving Street, NW, Washington, DC 20010, USA

## Abstract

**Background:**

Nearly half of all patients in the Intensive Care Unit (ICU) receive red blood cell (pRBC) transfusions (TFs), despite their associated complications. Restrictive transfusion strategy (Hemoglobin [Hb] < 7 g/dL) has been shown to reduce TF exposure. We estimated the potential annual reduction in transfusion-attributable severe acute complications (TSACs) and costs across the US ICUs with the adoption of restrictive strategy.

**Methods:**

A model, utilizing inputs from published studies, was constructed. Step 1 calculated potential number of patients appropriate for this strategy. In step 2, total number of pRBC units avoided with the restrictive trigger was extrapolated to the annual TFs in the US ICUs. Step 3 quantified excess acute complications and the number of pRBC units TF/1 TSAC in the TRICC trial. Step 4 transformed restrictive strategy-related avoidance of pRBC units to a reduction in TSACs, and step 5 quantified the associated cost savings.

**Results:**

Of the 4.4 million annual ICU admissions, 1,020,800 comprised the at-risk population. The total of 1,295,126 units of pRBC ($643/unit) could be saved with the restrictive strategy. Based on the data from the TRICC trial, dividing the 49 excess complications in the liberal group into the calculated excess of pRBCs transfused (1,624 units) yielded the rate of 33 pRBC units per one complication. Thus, dividing 1,295,126 units saved by 33 units/1 TSAC, the base-case analysis showed that 39,246 TSACs could potentially be avoided annually in the US ICUs, with the cost savings of $821,109,826.

**Conclusion:**

This model demonstrates that a restrictive transfusion strategy in appropriate at risk ICU patients is dominant and could result in improved quality of care and cost savings. Given the potential savings of 40,000 TSACs and nearly $1 billion, it is incumbent upon the intensivist community to promote more ubiquitous adoption of a clinically appropriate restrictive transfusion strategy in the ICU.

## Background

Allogeneic blood transfusion remains a cornerstone of therapy in the Intensive Care Unit (ICU), with nearly 50% of all ICU patients in the US and abroad receiving packed red blood cells (pRBCs) during their ICU course [1,2]. At the same time, a large body of evidence specific to the acutely ill hospitalized patients indicates that allogeneic blood is associated with an increased risk of infectious complications [3-5] and increased hospital length of stay [6]. Among the critically ill in particular, exposure to blood has been associated with a substantially increased risk of nosocomial infection [7-12], multiorgan failure [13], and death [14].

In addition to infectious complications, there are concerns about transfusion-related immunomodulation and a shrinking blood supply. Because of these issues, efforts have focused on ways to minimize an individual's exposure to pRBCs while in the ICU. One such effort was the TRICC trial [15], which showed that lowering of the transfusion trigger from 10 g/dL (liberal transfusion strategy) to 7 g/dL (restrictive transfusion strategy) in appropriate ICU patients results in comparable, and possibly even somewhat better, outcomes, with a substantial reduction in blood exposure and utilization. Notably there was a significant reduction in certain acute complications in the restrictive when compared to the liberal transfusion strategy group.

Unfortunately, recent data have indicated that utilization of the lower trigger is quite variable [1,2,16], particularly among patients on mechanical ventilation [17]. This indicates that we may be exposing the critically ill patients to potentially avoidable allogeneic blood, and may in turn be increasing their risk and overall number of transfusion-attributable severe acute complications (TSACs). Furthermore, since allogeneic blood carries an economic cost, there may be an opportunity to improve quality of ICU care while at the same time saving healthcare dollars via a restrictive transfusion strategy.

We sought to quantify the potential extent to which reducing the pre-transfision (pre-TF) hemoglobin (Hb) from the current practice levels to 7 g/dL, in appropriate patients, may result in the prevention of transfusion-attributable severe acute complications (TSACs). We additionally calculated the potential pRBC units and cost savings that may be realized with this reduction of overall utilization of blood. To address these questions, we constructed a simulation model based on recently published studies.

## Methods

No human subjects were enrolled in the study, and, thus, the study was exempt from regulations guiding protection of human subjects. This is a secondary analysis based on previously published and peer-reviewed data. All calculations were performed in Microsoft Excel. Multivariate simulations and sensitivity analyses were performed using Crystal Ball^® ^software (Decisioneering, Inc., Denver, CO).

### Model overview

We created an analytic model to determine the number of TSACs avoided annually in the US based on universal adoption of a restrictive transfusion strategy, defined as a transfusion trigger of < 7 g/dL [15]. Additionally, we estimated the potential number of units of pRBCs saved and the direct cost savings that would result from this reduction in blood utilization. The model was built from the perspective of a hospital.

### Model structure

**Figure 1:**
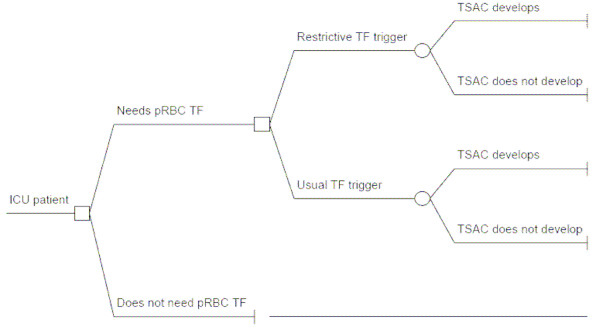
**Decision tree**. The square node at the left of the diagram represents the decision whether the patient requires a pRBC transfusion. The next decision node in the upper tree corresponds to the decision to pursue restrictive vs. usual current transfusion strategy. The circular chance nodes represent the probability of developing a TSAC with either strategy. The rightmost vertical segments are terminal nodes. *ICU, intensive care unit; pRBC, packed red blood cells; TF, transfusion; TSAC, transfusion-attributable sever complication

The model was designed to calculate the net difference in the frequency of TSACs in the US annually as a function of the decision to use lower (Hb < 7 g/dL) vs. current (Hb ~8.5 g/dL) transfusion trigger among patients determined to be at risk for a late transfusion (Figure 1). Late transfusions were defined as those required on and subsequent to ICU day #3 in the absence of active bleeding. We first determined the number of complications in a baseline scenario without the use of lower pre-TF Hb and then subtracted from that the number of complications in a situation where the trigger of < 7 g/dL was used uniformly. This equation (and other computations for this analysis) is illustrated in Table 1.

**Table 1:** Analytic framework for the model*

	**Model variable**	**Input**	**Source/Calculation**
A	Total number of ICU admissions annually in the US	4.4 mil	VERICC [18]
B	% Adult ICU admissions	0.8	Groeger 1993 [19]
C	% At risk for a late TF	0.29	Corwin 2002 [16]
D	Mean # units/pt at risk – current practice	3.01	Corwin 2002 [16]
E	Mean # units/pt TF – restrictive strategy group	2.6	Corwin 2002 [16]
F	Number of patients in restrictive group	418	Hebert [15]
G	% Patients transfused in restrictive group	0.67	Hebert [15]
H	Mean # units/pt at risk – restrictive practice	1.74	(fxgxe)/f
I	Total annual number of units TF – current practice	3,073,360	Axbxcxd
j	Total annual number of units TF – restrictive practice	1,778,234	Axbxcxh
k	Total annual number of units TF avoided restrictive practice	1,295,126	i-j
l	CV+ARDS SACs in liberal group	136	Hebert 1999 [15]
m	CV+ARDS SACs in restrictive group	87	Hebert 1999 [15]
n	TSACs	49	l-m
o	Total pRBC u TF liberal	2,352	Hebert 1999 [15]^¶^
p	Total pRBC u TF restrictive	728	Hebert 1999 [15]^¶^
q	pRBC u TF excess	1,624	o-p
r	Units/1 TSAC	33	q/n
s	TSACs avoided restrictive practice	39,246	k/r

*ICU, intensive care unit; TF, transfusion; rHuEPO, recombinant human erythropoietin; CV, cardiovascular; ARDS, adult respiratory distress syndrome; SAC, severe acute complication; TSAC, transfusion-attributable severe acute complication; pRBC, packed red blood cells; u, unit ^¶^The total number of units administered was calculated by multiplying the mean number of units transfused/patient by the number of patients transfused.

### Model inputs

The number of TSACs annually in the US can be computed based on 1) the estimated US ICU adult population considered at risk for a late transfusion, 2) the average number of pRBCs given to these individuals, and 3) the risk for a TSAC as a function of the number of pRBC units transfused. Tables 2, 3, 4 show our estimates for these variables and their sources in the literature. We relied on a recent epidemiologic survey [18] to approximate the US ICU population and then assumed 80% of these subjects were adults [19]. Since the TRICC trial utilized an artificially high TF trigger of 10 g/dL in the liberal arm, the data from this arm of the trial could not be used to estimate the extent of pRBC utilization in the current ICU practice environment. For this reason, transfusion practice and the amount of pRBCs given to an average ICU patient in the absence of a uniform utilization of a restrictive strategy was abstracted from the placebo arm of a recent randomized trial assessing the impact of recombinant human erythropoietin (rHuEPO) on transfusion utilization in critically ill subjects [16]. In this trial there was no uniform transfusion protocol, and the mean pre-TF Hb was 8.57 g/dL [16].

**Table 2:** Annual number of patients at risk for a late transfusion in US intensive care units

**Population variables**	**Input**	**Source**	**Output**
All US ICU admissions in 1 year	4,400,000	VERICC* [18]	4,400,000
% Adults	80%	Groeger [19]	3,520,000
% At risk for a late TF	29%	Corwin [16]	1,020,800

*VERICC, Values, Ethics and Rationing in Critical Care

**Table 3:** Transfusion-attributable severe acute complications and units packed red blood cells/1 transfusion-attributable severe acute complications derivation*

**Population variable**	**Input**	**Source**	**Output**
CV SAC			
Liberal	88 (21.0%)	Hebert 1999 [15]	
Restrictive	55 (13.2%)	Hebert 1999 [15]	
CV TSAC	88-55	Hebert 1999 [15]	33 (7.8%, 95% CI 2.7–12.9%)
ARDS SAC			
Liberal	48 (11.4%)	Hebert 1999 [15]	
Restrictive	32 (7.7%)	Hebert 1999 [15]	
ARDS TSAC	48-32	Hebert 1999 [15]	16 (3.8%, 95% CI–0.2–7.8%)
CV+ARDS TSAC	33+16		49
Total pRBC units TF liberal	5.6 u/pt x 420 pts TF	Hebert 1999 [15]	2,352
Total pRBC TF restrictive	2.6 u/pt x (418 x 0.67) pts TF	Hebert 1999 [15]	728
pRBC units TF excess	2,352–728		1,624
pRBC units/1 TSAC	1,624/49		33

* CV SAC, cardiovascular severe acute complication; CV TSAC, cardiovascular transfusion-attributable severe acute complication; ARDS SAC, adult respiratory distress syndrome severe acute complication; ARDS TSAC, adult respiratory distress syndrome transfusion-attributable severe acute complication

**Table 4:** Annual packed red blood cell transfusions avoided with restrictive transfusion strategy

**Population variables**	**Input**	**Source**	**Output**
Units pRBC TF/pt placebo arm	3.01	Corwin 2002 [16]	3.01
Units pRBC TF/pt restrictive group	1.74	Hebert [15]	1.74
Total units pRBC TF at risk ICU patients in 1 year			
Current practice	1,020,800 x 3.01		3,073,360
Restrictive practice	1,020,800 x 1.74		1,778,234
Total units avoided in 1 year with restrictive strategy	3,073,360–1,778,234		1,295,126
pRBC units/1 TSAC	1,624/49	Table 3	33
Total TSACs avoided	1,295,126/33		39,246

PRBC, packed red blood cells; TF, transfusion; pt, patient; TSAC, transfusion-attributable severe acute complication

To estimate the risk for TSACs we extracted additional data from the trial by Hebert et al. [15]. In this study subjects were randomized to either a restrictive transfusion strategy or a liberal approach. We defined as a TSAC all serious cardiovascular complications and cases of ARDS. We selected these endpoints from that trial as TSAC for two reasons: 1) they were objectively defined and recorded and 2) they essentially were shown to occur at different rates in the liberal and restrictive cohorts. More specifically, there were 33 fewer cardiovascular complications and 16 fewer instances of ARDS among those randomized to the restrictive approach (Table 3). We assumed that any difference in TSACs in the Hebert et al. trial reflected the results of differential exposure to pRBCs, and that the relationship between pRBC administration and TSACs followed a linear dose-response relationship (i.e., increasing risks for TSAC increased with greater exposure to pRBCs [11,12,20]). Dividing the total difference in TSACs into the calculated aggregate total difference in units of pRBCs administered between the two arms indicates that for every 33 units of pRBCs avoided, one TSACs is prevented (Table 3).

To calculate the number of TSACs avoided with the reduction of the transfusion trigger to 7 g/dL, it was first necessary to assess how many units of pRBCs might potentially be avoided with the universal use of 7 g/dL as the pre-TF Hb (Table 4). To evaluate this required two inputs: the proportion of US adult ICU patients who might be at risk for late transfusions, and the risk reduction accompanying change to a restrictive transfusion strategy in the ICU. Because the literature is vague on the number of patients remaining in the ICU to constitute the "at risk" group for late transfusions, we used the percentage of the screened patients who were deemed eligible for enrollment from the Corwin[16] study (29%). Next, we utilized the 3.01 units transfused/patient in the placebo group to calculate the overall blood utilization under current conditions in the patients at risk. Additionally, based on the experience of the TRICC investigators [15], we calculated that in the restrictive group the amount of transfused blood is on average 1.74 units across all the patients in the group. Multiplying this by the number of patients at risk yielded the total blood utilization under the circumstances of universal adoption of the restrictive transfusion practice. The difference between blood utilization under current conditions and that under universal use of restrictive practices was the potential number of pRBC units saved with the restrictive strategy institution (Table 4). As a final step, to calculate the potential number of TSACs avoided annually by using the Hb trigger of 7 g/dL, we simply divided the calculated number of units per 1 TSAC (n = 33) into the potential number of units saved with the restrictive strategy (Table 4).

### Cost assessment

Calculation of the cost savings associated with the reduced blood utilization was a simple product of the current cost of 1 unit of pRBC and the total number of units avoided. We determined the costs of a pRBC unit to be $634 from a report by Cremieux et al. [21] (year 2000 cost adjusted to 2005 US dollars based on the medical care services component for the Consumer Price Index).

### Monte Carlo simulations and sensitivity analyses

Because of uncertainty surrounding the point estimates for various model inputs, we conducted several Monte Carlo simulations and sensitivity analyses to assess the stability of these observations (Table 5). Monte Carlo simulation is a technique that allows resampling from the same population while varying several inputs simultaneously, thus resulting in random multivariate sampling of input variables across their corresponding ranges. We varied three inputs across a range of plausible values: the proportion of all adult ICU patients annually at risk for a late transfusion, the number of pRBCs required to precipitate one TSAC, and the cost of a unit of pRBC. Each of the outcomes, total TSACs avoided, total number of pRBCs saved and the cost savings associated with saving the number of pRBC units, was tested in 10,000 simulation trials, while simultaneously and randomly varying the three input values. Sensitivity analyses were performed to assess which input variables accounted for the largest amount of variation in the predicted values of the outcome variables.

**Table 5:** Sensitivity Analyses: Base case and upper and lower bound assumptions for the input variables*

Input variable	Base case assumption	Lower bound	Upper bound
% Adult ICU patients at risk for a late TF	29%^a^	13%^b^	50%^c^
Number of pRBC units/1 TSAC	33^d^	19^d^	552^d^
Cost per 1u pRBC	$634^e^	$250^f^	$800^f^

*pRBC, packed red blood cells; TF, transfused; TSAC, transfusion-attributable severe acute complication

^a^Based on % patients of the total pool screened deemed eligible for enrollment into Corwin study^16^

^b^Based on % patients of the total pool screened for the TRICC trial who gave their consent^15^

^c^Based on % patients of the total pool screened for the TRICC trial deemed eligible for enrollment^15^

^d^Based on 95% CI for the combine CV and ARDS complications from Hebert^15^

^e^Based on Cremieux^21^

^f^Based on anecdotal reports (see text for rationale)

For the sensitivity analyses, the lower and upper bounds for the input variables were derived as follows:

1. The proportion of all adult ICU patients annually at risk for a late transfusion were obtained from the TRICC trial (15):

a. Lower bound = percent of the patients of the total pool screened who gave their consent to participate in the trial (13%)

b. Upper bound = percent of all patients screened who were deemed eligible for the study

2. For the number of pRBCs required to precipitate one TSAC, the upper and lower bounds were obtained using the 95% confidence interval associated with the reduction in the risk of these events due to the restrictive transfusion strategy as compared to the liberal transfusion strategy in the TRICC trial (15). Specifically, the absolute difference in TSACs between the liberal and restrictive groups was adjusted to fall from a lower bound of 3 to an upper bound of 87. Thus one TSAC is avoided for between 552 units (where the effect of adopting a restrictive strategy would be the smallest – least effective) to 19 units (where the effect of adopting a restrictive strategy would be the greatest – most effective) of pRBCs transfused.

3. Because the cost of a unit of blood varies from region to region and tends to vary according to the underlying disease state (21), the upper and lower bounds of cost of a unit of pRBC were based largely on anecdotal reports of the cost of blood. To be inclusive, the lower bound used was $250 and the upper $800.

## Results

In our base-case scenario, and based on the inputs and assumptions described in the Methods section, we estimate that there are 1,020,800 adult subjects in ICUs in the US annually who may be at risk for a late transfusion Table 2). Utilizing current transfusion practices, these subjects receive 3,073,360 pRBC units. With the universal institution of a restrictive practice, the TF burden is reduced by 42% to 1,778,234 u pRBCs, resulting in a net savings of 1,295,126 units of pRBCs. Based on our estimated rate of TSACs in this population, this extent of transfusion avoidance prevents 39,246 additional TSACs annually, or approximately 1 TSAC per every 26 patients at risk for a late TF. Total annual blood management costs under current practice conditions are calculated to be $1,948,509,929, all due to the costs of pRBCs. With the restrictive strategy, the estimated cost savings realized would be $821,109,699 annually.

The base case, lower bound and upper bound values for the input variables in the simulations are shown in Table 5. After 10,000 trials for each outcome, the median values (95% confidence intervals) for annualized TSACs avoided, pRBCs saved and attendant cost savings were 39,246 (2,360 to 43,696), 1,295,126 (753,705 to 2,040,700), and $821,109,699 ($348,827,493 to $1,310,093,076), respectively. The worst case scenario was also examined. In the least effective scenario, where TSACs are infrequent (552 units pRBC/1 TSAC) and the lowest percentage of patients is at risk for late transfusions (13%), the number of TSACs avoided would be 1,052 annually. Conversely, in the most effective scenario, where TSACs are frequent (19 units pRBC/1 TSAC) and the highest percentage of patients is at risk for late transfusion (50%), there is a potential to avoid 84,137 TSACs.

While it is self-evident that the estimate of the proportion of ICU patients at risk for late transfusion contributed to 100% of the variability in the estimate of the number of pRBC units saved, it accounted for 59% of the variability in the estimated dollars saved, with the uncertainty around the cost of 1 unit of blood responsible for the remaining 41% of the variability. As for the variability in the estimates of the TSACs avoided, the single major contributor to this, with the 90% share, was the uncertainty in the estimate of the number of pRBC units associated with 1 TSAC; the remaining 10% of this outcome's variability was due to the uncertainty in the proportion of ICU patients at risk for a late pRBC transfusion.

## Discussion

This model-based simulation quantifies the magnitude to which the established literature suggests that TSACs are a significant burden in critically ill patients. Furthermore, our results indicate that efforts to prevent these TSACs through transfusion avoidance can enhance outcomes. Universal adoption of restrictive transfusion strategy represents one potential tool for transfusion avoidance. Our model underscores that a restrictive strategy may be a cost-saving, and thus dominant, option in an anemia management program, in addition to helping reduce the risk for the complications following pRBC transfusion.

Prior efforts to explore the relationship between transfusion and its negative sequelae in the ICU have focused on two areas: nosocomial infection and acute lung injury. Multiple observational analyses, both retrospective and prospective, have demonstrated a relationship between exposure to pRBCs and nosocomial infection. Taylor and colleagues, for example, in a review of their Project Impact database demonstrated an independent correlation between pRBC transfusion and nosocomial infections [8]. Addressing ventilator-associated pneumonia (VAP) specifically, Shorr et al. demonstrated a link between transfusion and this process [11], while Earley and co-workers confirmed a connection between transfusion and VAP in a cohort of trauma patients [20]. Recently Taylor et al. prospectively substantiated the nexus between transfusion and hospital-acquired infections [22].

Additionally, adverse pulmonary outcomes following transfusion are now better understood. Transfusion-related acute lung injury (TRALI) has emerged as a serious and underappreciated consequence of transfusion. Although estimated to occur in 1 in 5,000 transfusions, the incidence is likely higher in critically ill subjects where the condition may be difficult to distinguish from either the progression of previously underlying lung injury or other forms of pulmonary edema. Suggesting the existence of deleterious pulmonary complications of pRBC transfusions, Gong et al. reported that transfusion independently increased the risk for developing ARDS by 50% (adjusted odds ratio: 1.52, 95% CI: 1.00–2.31) in ICU patients with predisposing factors for ARDS [23]. Similarly, Kahn and colleagues in a group of individuals with subarachnoid hemorrhage noted that pRBC transfusion independently more than doubled the probability of ARDS [24]. Most recently another cohort study of nearly 5,000 critically ill patients found an adjusted odds ratio of 2.80 (95% confidence interval 1.90–4.12) of developing ARDS in association with pRBC transfusions and a dose-response relationship [25]. Finally, Gajic and coworkers have demonstrated an 18% absolute reduction in the development of acute lung injury in association with instituting a protocol limiting high tidal volume ventilation and inappropriate transfusions [26].

In light of these results, several systematic attempts have been made to limit the risks related to transfusion. Some advocate broader use of leukoreduction. Through leukoreduction, fewer pro-inflammatory mediators are infused into the recipient, and in turn, a less intense pro-inflammatory response arises. Despite theoretical benefits, it is unclear if leukoreduction will meaningfully decrease the rate of TSACs. In a large analysis of the Canadian experience with leukoreduction, post-transfusion fever was reduced, but there was no evident impact on nosocomial infection rates [27]. A recent randomized trial in acute trauma patients also failed to show any advantages of leukoreduction [28].

Recent efforts to limit ICU patients' exposure to allogeneic blood prompted a number of treatment studies with the rHuEPO. Thus, a 1999 study by Corwin et al. demonstrated a nearly 50% reduction in blood utilization, as well as a trend toward increased transfusion independence in the rHuEPO group [29]. A large Phase IIB follow-on trial by the same investigators found a 10% increase in transfusion avoidance and a 19% decrease in overall blood utilization [16]. Unfortunately, likely due to sample size, neither trial demonstrated a reduction in adverse events that may be attributable to exposure to allogeneic blood, or a favorable difference in other important outcomes. A large Phase III study was recently completed and should shed some light on the outstanding questions of how rHuEPO may fit into the blood management scheme in the ICU [30].

Reliance on a restrictive transfusion strategy, on the other hand, has appeared more successful at altering outcomes. As noted earlier, Hebert and colleagues showed that lower levels of hemoglobin are well tolerated in most critically ill patients, and that in some subjects fewer transfusions may be associated with improved outcomes [15]. Unfortunately, although some larger institutions appear to have successfully adopted a restrictive transfusion strategy [31], it is noteworthy that recent large European and US observational studies suggest that the transfusion trigger in most ICUs remains on average at approximately 8.5 g/dL (1, 2), and is actually even higher in the subgroup of patients on mechanical ventilation [17].

Why is it important to appreciate the extent of the burden of TSACs? An outcome has to be measured before it can be improved. This is most evident with blood stream infection [12] and ventilator-associated pneumonia [11,20,32], which have enjoyed improved outcomes through interventions aimed at their prevention [33,34]. Our study, by estimating their clinical and economic burden, sets the stage for focusing similar efforts to quantify and prevent some of the avoidable TSACs. An immediate action that may improve these outcomes is to adhere to the restrictive transfusion strategy whenever clinically feasible.

Our project's purpose was to determine to what extent universal adoption of a restrictive transfusion strategy might enhance efforts at transfusion avoidance and with what potential direct cost savings to the system. In that vein, our results show that a restrictive strategy may result in a substantial benefit to the patient and the system through avoidance of TSACs. For instance, we calculated that across-the-board utilization of a restrictive trigger would result in direct blood cost savings of $821 million annually. Given that ICU-acquired complications have substantial humanistic and economic costs [32], this strategy is likely to be even more attractive when one examines it in the context of ICU outcomes as a whole.

One prior report has attempted to detail the burden of blood-related complications in the ICU patients [35]. However, their calculations were based on estimates generated from epidemiologic data dealing with all subjects transfused, and not only those who are critically ill. As such, it is not clear that the results from reports dealing with populations of mixed severity of illness are generalizable to critically ill subjects. For example, TRALI arising in an otherwise mildly ill person is likely to have different consequences than TRALI in an already mechanically ventilated patient. Additionally, Shermock and co-workers only focused on several well-defined and rare transfusion-related complications [35]. They addressed viral transmission, bacterial contamination, and other similar processes that most clinicians would *a priori *associate with *de minimus *risk. Although possessing clear diagnostic criteria, these complications are likely not the major concerns of the intensivist. Hence, we sought to build and to expand on their efforts by exploring recently defined complications of transfusion that have been specifically described in critically ill patients.

In addition to focusing on outcomes that arise in the ICU, our analysis has several other strengths. We relied on data from recent reports describing the results of transfusion in critically ill patients. Moreover, most of the outcome inputs used in our model represent results of randomized, controlled trials [15,16]. Thus differences between those transfused and not transfused can be assumed to be directly attributable to added exposure to pRBCs. We also were conservative in our actual assumptions. Rather than assuming all subjects in an ICU might be at risk for a late transfusion, for example, we postulated that about 1/3 of all subjects might fit into this category. Furthermore, to formally evaluate the impact of uncertainty surrounding the point estimates for the model inputs, we conducted several sensitivity analyses to test the robustness of our conclusions. Finally, the model's transparency should aid in its applicability to concrete clinical situations.

Nonetheless, our study has several important limitations. First, despite our attempt to acknowledge and adjust for uncertainty, the data from which we draw our model inputs are limited. Unfortunately there are relatively few well-done studies exploring transfusion epidemiology, potential adverse events related to transfusion in the critically ill, and the significance of adoption of restrictive transfusion strategy in the ICU in the real world. Studies quantifying the actual cost of blood in the ICU setting are scarce as well. For this reason our final sensitivity estimates are relatively broad and imply intrinsic sensitivity to the accuracy of the input variables' values. That is, the most significant influence on the number of TSACs avoided in the least effective scenario is the assumption that it takes 552 units to precipitate 1 TSAC. While this number is based on the 95% confidence intervals reported by Hebert [15], it needs to be viewed in the context of observational studies that suggest a much greater incremental risk associated with exposure to each unit of pRBCs [8,11,12]. Second, we assumed that the incidence of our primary outcomes of interest, cardiovascular complications and ARDS/ALI, follows a linear dose-response relationship with pRBC exposure. Although some observational studies suggest this to be the case [11,12,22], to the best of our knowledge, no well-designed prospective study has been carried out to confirm this. In reality, both of these adverse outcomes might not be linearly related to pRBC transfusion amounts, but could alternatively follow a threshold effect. Third we did not add the economic or clinical cost of an actual TSAC into our model due to the paucity of such data in peer-reviewed literature. Fourth, the data on liberal transfusion-associated complications are derived from a single trial [15]. This may be problematic in several ways: 1). Risk estimates from a trial may not reflect accurately the risks in real practice, and 2). Given a vast list of exclusion criteria and treating physician's discretion with respect to the decision to enroll patients, the results may not be generalizable to all ICU patients. Since a similar study is not likely to be replicated in subpopulations of patients of interest, we have attempted to overcome these short-comings by performing sensitivity analyses utilizing fairly conservative risk estimates and thus biasing the model against restrictive strategy. However, the possibility remains that by applying the strategy across the board, our model may thus overestimate the true effect that the institution of a sensible restrictive practice appropriate to a multitude of clinical situations would have. Finally, the sources for some of the critical inputs were derived from two different trials with potentially different populations. To assess this, we examined the potential differences between the restrictive strategy group in the TRICC trial and the placebo arm in the Corwin study (Table 6) [15,16]. Although the placebo group in the Corwin trial (n = 652) was slightly larger than the restrictive arm in the TRICC study (n = 418), they were similar in their mean age, gender distribution and APACHE II scores. As for primary diagnoses, there was an imbalance between the groups in the proportion of patients with trauma, 20% vs. 48.5%, and cardiovascular disease, 18% vs. 5.4%, in TRICC and Corwin, respectively. While a deficit of cardiovascular diagnoses may have biased the Corwin placebo arm in the direction of a restrictive strategy, the excess of trauma patients would operate in the opposite direction, potentially biasing against a restrictive strategy. However, these limitation notwithstanding, to the best of our knowledge, ours is the first study to estimate the plausible range of avoidable costs and complications is restrictive transfusion strategy were to be adopted more uniformly in the ICUs across the US.

**Table 6:** This table depicts pertinent baseline characteristic and selected admitting diagnoses of patients enrolled into the restrictive arm of the TRICC trial [15] and those in the placebo arm of the randomized controlled trial of rHuEPO treatment of critically ill patients [16], the groups from which our main input parameters were derived*.

	**Restrictive strategy, TRICC (N = 418)**	**Placebo, rHuEPO RCT (n = 652)**
Age, mean ± SD, years	57.1 ± 18.1	51 ± 19.4
Gender male	64%	63.7%
APACHE II score, mean ± SD	20.9 ± 7.3	19.6 ± 7.99
Proportion post-operative	39%^a^	42.5%
Proportion trauma	20%	48.5%
Proportion sepsis/SIRS	6%	7.7%^b^
Proportion respiratory disease	28%	25%^c^
Proportion cardiovascular disease	18%	5.4%

*TRICC, Transfusion Requirements in Critical Care^15^; rHuEPO, recombinant human erythropoietin; RCT, randomized controlled trial; SD, standard deviation; APACHE, Acute Physiology and Chronic Health Evaluation; SIRS, systemic inflammatory response syndrome.

^a^This is the number corresponding to proportion of patients with location before ICU admission being the operating room or the recovery room^15^

^b^This was calculated by adding together the proportion of sepsis and SIRS patients^16^

^c^This was calculated by adding together the proportion of patients with pneumonia, ARDS and other respiratory diagnoses^16^

## Conclusion

Our model estimates that universal adoption of a restrictive transfusion strategy could result in avoidance of nearly 40,000 transfusion-attributable severe acute complications annually among the critically ill in the US. This in turn could result in the savings of nearly $1 billion in costs associated with blood alone to the healthcare system. Furthermore, these findings remain robust across a range of sensitivity analyses. Efforts should be undertaken to support more ubiquitous adoption of a clinically appropriate restrictive transfusion strategy in the ICU.

## Abbreviations

ICU, intensive care unit; pRBC, packed red blood cells; TF, transfusion; TSAC, transfusion-attributable sever acute complication; ARDS, acute respiratory distress syndrome; rHuEPO, recombinant human erythropoietin; VAP, ventilator-associated pneumonia; TRALI, transfusion-related acute lung injury; CI, confidence interval; ALI, acute lung injury

## Competing interests

Dr. Shorr has served as a consultant to Ortho Biotech Clinical Affairs, LLC, manufacturer of epoetin alfa. Dr. Zilberberg is a former employee of and has served as a consultant to Ortho Biotech Clinical Affairs, LLC. She is also a stockholder in Johnson & Johnson, its parent company.

## Authors' contributions

MDZ carried out study design, analysis, data interpretation, drafting the manuscript. AFS carried out study design, data interpretation, drafting the manuscript. Both authors read and approved the final manuscript.

## Pre-publication history

The pre-publication history for this paper can be accessed here:


